# Impact of Medication for Opioid Use Disorder on Patient Directed Discharge Among Patients with Opioid Use Disorder

**DOI:** 10.1007/s11606-026-10172-5

**Published:** 2026-01-23

**Authors:** Sumeet Singh-Tan, Andrea Jakubowski, Zina Huxley Reicher, Kristine Torres-Lockhart, Jeffrey Ceresnak, Jessica Pacifico, Julia Arnsten, William Southern

**Affiliations:** 1 Department of Medicine, Division of Hospital Medicine, Albert Einstein College of Medicine, Montefiore Medical Center, Bronx, NY USA; 2 Department of Medicine, Division of General Internal Medicine, Albert Einstein College of Medicine, Montefiore Medical Center, Bronx, NY USA; 3 Department of Medicine, Psychiatry and Behavioral Sciences, Albert Einstein College of Medicine, Montefiore Medical Center, Bronx, NY USA; 4 Department of Medicine, Epidemiology and Population Health, Albert Einstein College of Medicine, Montefiore Medical Center, Bronx, NY USA

**Keywords:** opioid use disorder, substance use disorders, patient-directed discharge, hospitalization, medications for opioid use disorder (MOUD)

## Abstract

**Background:**

Opioid use disorder (OUD) is responsible for significant morbidity and mortality in the USA. Hospitalization rates for patients with OUD have increased over the recent decades. Those with OUD have a substantially higher rate of patient-directed discharge (PDD) than those without OUD. There have been mixed results when examining the association between inpatient MOUD and PDD.

**Objective:**

To determine the association between inpatient MOUD and the rate of PDD among patients without evidence of MOUD treatment prior to hospitalization.

**Design:**

Retrospective study comparing admissions receiving inpatient MOUD and propensity score–matched control admissions who did not receive MOUD.

**Subjects:**

Two thousand seven hundred seventy-one admissions with a diagnosis of OUD and without evidence of prior MOUD treatment were compared to 2771 propensity-matched admissions.

**Intervention:**

Provision of inpatient MOUD, either buprenorphine or methadone during admission.

**Main Measures:**

Primary outcome was patient-directed discharge. Secondary outcomes were buprenorphine prescription at discharge, buprenorphine prescription within 60 days of discharge, admission into an outpatient methadone program within 30 days of discharge, 30-day readmission, and 30-day post-discharge ED visit.

**Key Results:**

Among 5542 admissions with OUD and no evidence of MOUD prior to admission, those that received inpatient MOUD were significantly less likely to have a PDD (11.9% vs 14.4%; OR 0.80 [CI 0.67–0.96]) and significantly more likely to receive a discharge prescription for buprenorphine (8.6% vs 1.2%; OR 8.04 [CI 5.52–11.71]) and another buprenorphine prescription within 60 days of discharge (5.5% vs 1.1%; OR 5.09 [CI 3.35–7.74]), compared with control admissions who did not receive MOUD. Inpatient MOUD was not significantly associated with admission into an outpatient methadone program within 30 days, 30-day readmission, and 30-day post-discharge ED visit.

**Conclusions:**

Receipt of inpatient MOUD was associated with a statistically significant reduction in PDD among those with OUD and without evidence of MOUD before admission when compared with propensity-matched admissions which did not receive inpatient MOUD.

**Supplementary Information:**

The online version contains supplementary material available at 10.1007/s11606-026-10172-5.

## INTRODUCTION

Opioid use disorder (OUD) affects 2.5 million people in the USA and was responsible for nearly 73,000 deaths from overdose in 2023.^1^ People with OUD have ten-times greater mortality than those without OUD.^2^ Hospitalization rates for those with OUD have increased threefold since 1998.^3^ A study by Thakrar et al. found that outcomes during hospitalization also differ between patients with and without OUD in that patients hospitalized with a diagnosis of OUD have a patient-directed discharge (PDD) rate of 12%. This rate is even higher (18%) among those admitted with an injection-related infection. These PDD rates are dramatically higher than the 1% rate for patients without OUD.^4^ Patients leaving the hospital before medically advised have a twofold increase in their 30-day mortality risk compared to those discharged when medically recommended^5^.  Additionally, PDD among patients with OUD is associated with fragmented post-discharge care, increased readmission, and increased mortality compared to planned home-discharges.^6^

There are three FDA-approved medications for opioid use disorder (MOUD): methadone, buprenorphine, and intra-muscular naltrexone. Methadone and buprenorphine are often favored in hospital settings because they can be initiated relatively quickly and do not require a significant waiting period for withdrawal from opioids as naltrexone does. These two medications have also been shown to be effective at reducing opioid-related and all-cause mortality,^7,8^ reducing infectious complications of opioid use,^9^ reducing withdrawal symptoms,^10^ and increasing outpatient care.^11^ Previous study has shown that undertreatment of withdrawal is among several factors influencing PDD among patients with OUD and other substance use disorders.^12^ While several studies have examined the effect of MOUD on PDD, they have been limited by inclusion of specific cohorts,^13–15^ a single MOUD,^16^ not distinguishing between those receiving or not receiving MOUD at time of hospitalization,^17^ and small sample size.^14,18,19^ Due to the heterogeneity of these studies, they have yielded mixed results with some showing MOUD-associated reductions in PDD and others showing no difference.

Treating hospitalized patients with MOUD has the potential to reduce rates of PDD and may also improve longer-term health outcomes, such as MOUD continuation and reductions in readmissions and ED visits. We hypothesized that hospitalized patients with OUD who receive MOUD during admission have a lower rate of PDD compared to those not receiving MOUD. We also posited that there may be improved outcomes related to continuing MOUD treatment post-discharge and reductions in ED visits and readmissions.

## METHODS

### Study Setting and Design

Montefiore Medical Center consists of three academic hospitals in Bronx, NY. It serves a primarily Hispanic/Latine and non-Hispanic Black community in one of the nation’s poorest urban counties.^20^ During the study period (January 1, 2016–December 31, 2022), two hospitals had interprofessional addiction consult services^21^ which differed in staffing (addiction psychiatry vs addiction medicine; social worker vs peer outreach specialists), availability (weekdays vs 6 days/week), and year established (2015 vs 2021). We aimed to evaluate the impact of receiving inpatient MOUD, regardless of addiction consultation, on the risk of PDD and other outcomes among admitted patients with OUD for whom there was no evidence of MOUD treatment prior to admission. To address the potential for confounding by indication, wherein patients who receive inpatient MOUD would have differing characteristics than those who did not, we used a propensity score–matched study design.^22^ To create the matched study sample, each admission that received inpatient MOUD was matched with a control admission that did not receive MOUD using a propensity score matching protocol further described below. This study was determined to be exempt by the Albert Einstein College of Medicine Institutional Review Board.

### Study Population

We included all admissions with a diagnosis of OUD based on International Classification of Diseases (ICD-10) code assigned at discharge (supplement 1), without evidence of treatment with MOUD before admission and that were discharged between January 1, 2016, and December 31, 2022. Because many patients had more than one admission meeting the study criteria, and each individual admission was an opportunity for intervention, we use *admission* rather than *patient* to refer to the unit of analysis. Evidence of treatment with MOUD before admission was defined as: methadone dose > 40 mg by day 2 of admission or evidence of enrollment in a Montefiore-affiliated treatment site within the 60 days prior to admission or buprenorphine dose > 2 mg on day 1 of admission or buprenorphine prescription within the 60 days prior to admission. Our definition of prior MOUD treatment considered that Montefiore is a major provider of MOUD in the Bronx (five of 13 opioid treatment programs in the Bronx are Montefiore affiliated),^23^ however, only considering outpatient treatment programs would not capture all admissions with prior methadone treatment since eight of the opioid treatment programs are not within the Montefiore network. Because of this, a 40 mg methadone dose by day 2 of admission was also used since most people enrolled in a methadone treatment program receive a dose greater than 40 mg and chart review showed that it often takes 2 days to verify and order their outpatient treatment dose with their outpatient program. Lastly, during the study period, there was an institutional practice of limiting methadone doses to 40 mg for patients whose enrollment in an outpatient program could not be verified with a phone call. A 1-day inpatient cutoff was used for buprenorphine since quicker verification was possible with a patient’s pharmacy or with the state prescription monitoring program. We excluded dates when COVID-19 was prevalent in the hospital because of its significant effect on inpatient care and outcomes (supplement 2).

### Intervention

We determined the impact of inpatient treatment with MOUD on PDD. Inpatient MOUD was defined as treatment with any dose of methadone or buprenorphine during admission. Naltrexone was excluded from the study as its requirement of opioid abstinence for several days often makes it an unfavorable option in the inpatient setting.

### Measures

#### Outcomes

The primary outcome was PDD. This was defined by a disposition code of discharge against medical advice or elopement in the medical record. Because prior studies have shown that patients who receive MOUD are more likely to remain engaged in care after hospitalization,^11^ we examined several secondary outcomes including buprenorphine prescription at discharge, buprenorphine prescription within 60 days of discharge, admission into an outpatient methadone program within 30 days of discharge, 30-day readmission, and 30-day post-discharge ED visit. Buprenorphine prescription at discharge was defined as a prescription for the medication written at hospital discharge. Buprenorphine prescription within 60 days of discharge was defined as having an electronic medical record prescription within 60 days of discharge. Admission to an opioid treatment program within 30 days of discharge was defined as a new completed admission to a Montefiore-affiliated treatment program. Because Montefiore has a large network of outpatient treatment programs and most patients are referred to a network treatment center at discharge, we believed the vast majority of referrals to outpatient treatment would be captured. 30-day readmission and post-discharge ED visit were dichotomous outcomes defined as a hospital readmission or ED visit within the Montefiore Medical Center during the 30 days after discharge.

#### Covariates

To characterize the study population and create propensity-matched cohorts, we examined the demographic, clinical, and hospitalization characteristics for each admission. Demographic characteristics included age, sex, race/ethnicity (categorized into four groups for analysis: Black/African American, Latine, non-Hispanic White, and other/unknown), and insurance (categorized into five groups: Medicaid, Medicare, commercial, other, and self-pay). Clinical characteristics included the following ICD-10 diagnosis codes assigned at discharge: OUD diagnosis type (categorized into five groups based on ICD-10 distinctions: poisoning, opioid abuse and opioid-induced disorder, opioid use and opioid dependence with complications, opioid use with adverse effect, and opioid use and opioid dependence without complication), presence of a co-occurring substance use disorder (including cocaine, sedative/hypnotic, stimulant, or alcohol use), presence of a co-occurring mental health disorder, presence of infection (bacteremia, cellulitis, or osteomyelitis), presence of liver disease. Clinical characteristics were also determined by the Charlson comorbidity index (a dichotomous variable indicating a score less than two or greater than or equal to two), and the number of admissions and ED visits in the year prior to admission (categorized into four groups: no admissions/ED visits, 1–4 admissions/ED visits, 5–9 admissions/ED visits, and ≥ 10 admissions/ED visits). The hospitalization characteristics were admission campus (one of three), discharge from the medicine service (a dichotomous variable indicating medicine discharge or discharge from any other service), admission to the intensive care unit (ICU) from the ED, and receipt of an addiction consult service consultation (all dichotomous variables).

### Creation of Matched Cohorts

For each admission that received inpatient MOUD, we searched for a propensity-matched control admission using all demographic, clinical, and hospitalization characteristics noted (Table 1). Matching for the intervention and control cohorts was done using a nearest neighbor protocol with a caliper width of 0.05 of the standard deviation of the logit of the propensity score with replacement, with a prespecified 1:1 matching ratio.

**Table 1:** Admission Characteristics of Propensity-Matched Cohorts

	Inpatient MOUD (*n* = 2771)	Matched control (*n* = 2771)	*p***-**value	Standardized difference
Demographic				
Age, mean (SD)	54.7 (12.3)	54.5 (13.9)		0.02
Male sex, %	1629 (58.8%)	1612 (58.2%)	0.64	0.01
Race/ethnicity, %			0.65	0.01
Black/African American (non-Hispanic)	739 (26.7%)	716 (25.8%)		
Hispanic/Spanish/Latinx	1337 (48.3%)	1368 (49.4%)		
White (non-Hispanic)	376 (13.6%)	389 (14.0%)		
Other race^*^	319 (11.5%)	298 (10.8%)		
Insurance, %			0.53	0.01
Medicaid	1693 (61.1%)	1722 (62.1%)		
Medicare	936 (33.8%)	897 (32.4%)		
Commercial	129 (4.7%)	141 (5.1%)		
Other	11 (0.4%)	7 (0.3%)		
Self pay	2 (0.1%)	4 (0.1%)		
Clinical				
ICD-10 OUD diagnosis, %^†^			0.94	0.001
Poisoning	190 (6.9%)	179 (6.5%)		
Opioid abuse and opioid-induced disorder	312 (11.3%)	312 (11.3%)		
Opioid use and opioid dependence w/complication	404 (14.6%)	423 (15.3%)		
Opioid use with adverse effect	307 (11.1%)	308 (11.1%)		
Opioid use and opioid dependence w/o complication	1558 (56.2%)	1549 (55.9%)		
Other SUD^§^, %	821 (29.6%)	788 (28.4%)	0.33	0.03
Mental health ICD-10 diagnosis	1428 (51.5%)	1412 (51.0%)	0.67	0.01
Bacteremia, %	29 (1.1%)	33 (1.2%)	0.61	0.01
Cellulitis, %	246 (8.9%)	235 (8.5%)	0.60	0.01
Osteomyelitis, %	101 (3.6%)	83 (3.0%)	0.18	0.04
Charlson comorbidity index ≥ 2, %	1752 (63.2%)	1741 (62.8%)	0.76	0.01
Liver disease^†^, %	727 (26.2%)	720 (26.0%)	0.83	0.01
Previous ED visits (1 year), %			0.91	< 0.01
None	685 (24.7%)	669 (24.1%)		
1–4 visits	1368 (49.4%)	1382 (49.9%)		
5–9 visits	371 (13.4%)	382(13.8%)		
≥ 10 visits	347 (12.5%)	338 (12.2%)		
Previous admissions (1 year), %			0.64	< 0.01
None	1185 (42.8%)	1161 (41.9%)		
1–4 admissions	1144 (41.3%)	1183 (42.7%)		
5–9 admissions	293 (10.6%)	293 (10.6%)		
≥ 10 admissions	149 (5.4%)	134 (4.8%)		
Hospitalization				
Hospital, %			0.90	0.01
Hospital 1	1519 (54.8%)	1534 (55.4%)		
Hospital 2	502 (18.1%)	501 (18.1%)		
Hospital 3	750 (27.1%)	736 (26.6%)		
Medicine service, %	2132(76.9%)	2110 (76.2%)	0.49	0.02
ICU admission, %	180 (6.5%)	176 (6.4%)	0.83	0.01
ACS consult, %	74 (2.7%)	78 (2.8%)	0.74	0.01

^*^Includes Asian, Native American/Alaskan Native, and race not available

^†^Based on ICD-10 diagnosis code listed at discharge

^§^Includes cocaine, sedative/hypnotic, stimulant, and alcohol use disorders based on ICD-10 diagnoses

all above variables were added to the propensity score model

### Analysis

First, the demographic, clinical, and hospitalization characteristics of the matched cohorts were compared using *t*-tests and chi-square tests as appropriate. Next, to determine the associations between inpatient MOUD and the outcomes (patient-directed discharge, buprenorphine prescription at discharge, buprenorphine prescription at 60 days, admission to outpatient methadone program within 30 days of discharge, 30-day readmission, and 30-day post-discharge ED visit), we used logistic regression models. Because the unit of analysis was a hospital admission, all regressions used cluster-robust standard errors to account for the clustering of multiple admissions within individual patients. We planned to use multivariable logistic regressions to adjust for variables that were unbalanced between the matched cohorts with a standardized mean difference greater than 0.1; however, all covariates were balanced after matching, so no multivariable adjustment was performed. Finally, subgroups for analysis were determined a priori based on characteristics investigators theorized might be indicative of more severe OUD and greater health risk: comorbid mental health disorder,^24^ elevated Charlson comorbidity index,^25^ comorbid substance use disorder,^24,25^ discharge diagnosis of opioid poisoning, presence of liver disease,^26^ initial admission to the ICU,^27^ and presence of infection.^28^ A separate univariable regression model examined the association between inpatient treatment and outcomes in each subgroup. Wald statistic *p*-values for interaction were calculated using regression models which included interaction terms. All analyses were done using Stata 17.0 software (College Station, TX).

## RESULTS

### Study Population

**Figure 1:**
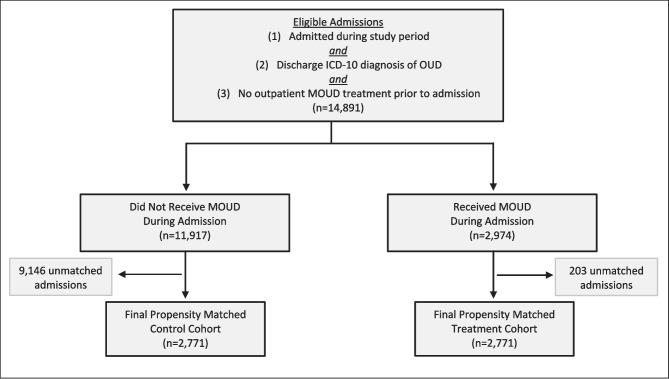
Flow diagram for propensity-matched admissions.

There were 14,891 admissions eligible for the study (representing 9410 unique patients). Of these, 2974 admissions received inpatient MOUD treatment. Of the admissions receiving treatment, 2771 were successfully matched with a control admission that did not receive inpatient MOUD, yielding a final study sample of 5542 admissions (Fig. 1). Cohorts were similar with respect to demographic, clinical, and hospitalization characteristics, as noted by standardized mean differences less than 0.1 (Table 1). Of the 2771 admissions that received treatment, the majority, 81.2%, received methadone (Table 2).

**Table 2:** MOUD Given

Methadone	2251 (81.2%)
Buprenorphine	442 (16.0%)
Both	78 (2.8%)

### Patient-Directed Discharge

Admissions receiving inpatient MOUD were significantly less likely to have a PDD when compared with admissions not receiving inpatient MOUD (11.9% vs 14.4%; OR 0.80 [CI 0.67–0.96]) (Table 3). The subgroup analysis showed that there was a greater benefit of inpatient MOUD among admissions with a comorbid mental health diagnosis (OR 0.72 [CI 0.56–0.72]) compared to those without a comorbid mental health diagnosis (OR 0.89 [CI 0.70–1.12]), but the difference did not reach statistical significance (*p* for interaction 0.21). Similarly, there was a greater benefit among those admitted without an infection (OR 0.75 [CI 0.62–0.92]) compared to those admitted with an infection (OR 1.02 [CI 0.69–1.50]); however, this difference did not reach statistical significance (*p* for interaction 0.17). Our other subgroup analyses did not reveal significant differences of MOUD treatment among those with higher Charlson comorbidity scores, comorbid substance use disorders, comorbid liver disease, or an admission to the ICU (Table 4).

**Table 3:** Outcomes

	Inpatient MOUD (*n* = 2771)	Matched control (*n* = 2771)	*p*-value	Odds ratio (CI)
Primary				
Patient-directed discharge	329 (11.9%)	399 (14.4%)	0.005	0.80 (0.67–0.96)
Secondary				
Buprenorphine prescription at discharge	238 (8.6%)	32 (1.2%)	< 0.001	8.04 (5.52–11.71)
Buprenorphine prescription at 60 days	151 (5.5%)	31 (1.1%)	< 0.001	5.09 (3.35–7.74)
Admission to outpatient methadone w/in 30 days	32 (1.2%)	36 (1.3%)	0.63	0.89 (0.54–1.47)
Post-discharge ED visit (30 d)	868 (31.3%)	873 (31.5%)	0.89	0.99 (0.85–1.12)
Readmission (30 d)	678 (24.5%)	677 (24.4%)	0.98	1.00 (0.85–1.18)

**Table 4:** Patient-Directed Discharge by Subgroups

	OR (95% CI)	*p* for interaction
Comorbid mental health diagnosis		0.21
Absent	0.89 (0.70–1.12)	
Present	0.72 (0.56–0.92)	
Charlson comorbidity index		0.51
< 2	0.75 (0.59–0.96)	
≥ 2	0.85 (0.66–1.10)	
Comorbid SUD		0.36
Absent	0.73 (0.59–0.92)	
Present	0.86 (0.66–1.11)	
Opioid poisoning		0.34
Absent	0.82 (0.68–0.98)	
Present	0.60 (0.32–1.11)	
Liver disease		0.93
Absent	0.80 (0.66–0.98)	
Present	0.79 (0.56–1.12)	
ICU admission		0.92
No	0.80 (0.67–0.96)	
Yes	0.83 (0.43–1.59)	
Infection		0.17
Absent	0.75 (0.62–0.92)	
Present	1.02 (0.69–1.50)	

### Secondary Outcomes

Admissions receiving inpatient MOUD were significantly more likely to receive a discharge prescription for buprenorphine when compared with those not receiving inpatient MOUD (8.6% vs 1.2%; OR 8.04 [CI 5.52–11.71]). There was also a statistically significant increase in buprenorphine prescriptions within 60 days after hospital discharge among those receiving inpatient medication (5.5% vs 1.1%; OR 5.09 [CI 3.35–7.74]). Receiving inpatient medication did not have statistically significant associations with new enrollment in a Montefiore outpatient treatment program within 30 days of discharge (1.2% vs 1.3%; OR 0.89 [CI 0.54–1.47]), 30-day post-discharge ED visits (31.3% vs 31.5%; OR 0.99 [CI 0.85–1.12]), nor 30-day readmissions (24.5% vs 24.4%; OR 1.00 [CI 0.85–1.18]) (Table 3).

## DISCUSSION

In a carefully controlled propensity score–matched study, we found that among admitted patients with a diagnosis of OUD, for whom there is no evidence of MOUD treatment prior to admission, inpatient MOUD is associated with a significant reduction in patient-directed discharge as well as a significant increase in discharge prescription for buprenorphine. This is the first study to our knowledge to find such an association among patients without evidence of prior MOUD.

A prior study comparing admissions of those receiving MOUD prior to hospitalization and those who were not found that patients receiving MOUD before admission had a significant reduction in PDD when given inpatient MOUD. This association was not seen among patients who were newly initiated on MOUD during their admission.^16^ This finding suggests that not receiving MOUD prior to hospital admission may be a particularly high-risk characteristic as it relates to PDD. Another study examining PDD rates among patients assigned to one of four interventions found that those receiving MOUD during admission were less likely to have a PDD when compared to the other interventions, which included receiving only short-acting opioids, receiving only non-opioid adjunctive medications to manage withdrawal symptoms, or no medication at all^17^. Although this study found a benefit in providing MOUD, it did not distinguish between those who were already receiving MOUD at admission and those who were not. These patients have differing characteristics and likely different factors influencing their healthcare decision-making, including their decision to remain in the hospital for medical treatment. Our study only included admissions that did not show evidence of prior MOUD treatment because of their presumed higher risk and found a statistically significant reduction in PDD among those who were given MOUD. This difference in result may be explained by our propensity-matched design, which allowed for more similar comparison groups among those who did and did not receive MOUD.

Our subgroup analyses did not reveal statistically significant differences among the subgroups examined; however, they did reveal a benefit of treatment among those with a comorbid mental health diagnosis. Because a prior study has shown that those with OUD have a higher prevalence of mental health diagnoses,^29^ there is potential for particularly meaningful impact of MOUD in this population as there was a strong association between MOUD and a reduction in PDD among those with a comorbid mental health diagnosis. Additionally, there was an indication of greater benefit of inpatient MOUD among those without a diagnosis of infection (bacteremia, cellulitis, osteomyelitis). This is an encouraging area for further study as a prior study of this population has yielded mixed results with some studies showing a reduction in PDD with MOUD initiation^13,15^ and another showing no difference.^14^ Although the differences in the impact of MOUD on PDD did not reach statistical significance in all subgroups, there was signal indicating that less severe disease (lower Charlson Comorbidity index, no comorbid substance use disorder, absence of opioid poisoning diagnosis, absence of liver disease, non-ICU admission, and absence of infection) was associated with greater benefits of MOUD. Taken together, these findings suggest that there may be a benefit to providing MOUD to patients with less severe comorbidity to have a greater impact on patient-directed discharge.

We found that inpatient MOUD was associated with an increased odds of buprenorphine prescription at discharge as well as a refill prescription within 60 days following discharge. This is consistent with prior studies^30,31^ showing inpatient MOUD was associated with increased linkage to MOUD after discharge. Of note, the study period took place during the requirement of a DEA-X waiver to prescribe buprenorphine. This greatly limited the number of providers who were able to prescribe buprenorphine at discharge. This limitation likely accounts for the overall low number of buprenorphine prescriptions at discharge (8.6%). Our study did not find that inpatient MOUD was associated with an increase in outpatient methadone treatment program enrollment within 30 days. The difference in outcomes related to buprenorphine and methadone may be because outpatient methadone treatment referral requires a significant amount of care coordination, whereas buprenorphine requires only a prescription which can be written by any licensed medical provider with a DEA number. Further, methadone treatment for the indication of OUD can only be dispensed from a specific clinical facility, whereas buprenorphine can be procured at any pharmacy. A prior study showed that consultation with an interdisciplinary addiction consult service is associated with increased uptake of outpatient methadone treatment 30 days after discharge.^32^ This finding highlights the importance of consultative care in navigating continued MOUD after discharge, specifically with methadone, and that inpatient MOUD treatment alone is likely insufficient to achieve continued post-discharge treatment with this medication which requires completion of an intake appointment and daily appointments for obtaining methadone. These barriers likely account for the overall low admission into a methadone treatment program.

We further found that inpatient MOUD was not associated with reductions in 30-day readmissions or 30-day ED visits. This is similar to findings of prior studies.^19,32^ This may be related to the underlying degree of healthcare need due to the severity of OUD as well as underlying medical comorbidities among patients admitted with OUD. Our study sample showed that 16% of admissions had at least 5 admissions over the prior year and 26% had at least 5 ED visits over the prior year. Further, 63% of admissions had a Charlson comorbidity index of two or greater. These factors indicate the severity of illness of the study population and point to the high level of medical need often present in patients with OUD. It also highlights the potential benefit of earlier treatment engagement when medical conditions are less severe. These findings illustrate the complexity of OUD and show that clinically meaningful impact likely requires many points of contact over time.

Our study has several limitations. Firstly, it was a single center observational study, which may limit the generalizability to other settings. In particular, the demographic characteristics of the Bronx-based study population may not be similar to many practice settings. Secondly, all data are sourced from the Montefiore Medical Center treatment network, which limits findings to those who sought care within this system alone. If patients sought care outside of the Montefiore network, those findings would not have been captured in this study. However, because the medical center is a major provider of MOUD and general medical care in the Bronx, we feel confident that our study findings are not spurious and accurately reflect the benefits of inpatient MOUD. Additionally, the possibility of confounding remains due to uncollected and unmeasured variables in this observational study. Further, we used discharge ICD-10 diagnoses to determine the presence of OUD and to determine the presence of included comorbidities. This method leaves a risk for misclassification because several conditions may be underdiagnosed or inaccurately assigned.^33^

An important strength of our study was our propensity-matched control design, which allowed for more comprehensive confounding factor adjustment. Another strength is that it describes outcomes in a clinical setting that serves many Black and Latine patients who historically have had unequal access to MOUD.^34^ Our study further emphasizes the importance of inpatient MOUD to reduce rates of PDD and increase linkage to outpatient MOUD for Black and Latine patients who have historically been underserved.

## CONCLUSION

Overall, our study findings provide evidence of the benefit of inpatient MOUD for patients with OUD who have not received OUD treatment prior to admission, specifically in reducing patient-directed discharge and increasing buprenorphine prescribing and continuation after discharge. The prevalence of OUD has increased in recent years, as have hospitalizations of patients with OUD. These patients often experience high clinical complexity and significant social challenges. This study supports the potential benefit of providing inpatient MOUD, which has the potential to increase healthcare participation, improve health outcomes, and continue engagement after discharge.

## Supplementary Information

Below is the link to the electronic supplementary material.

ESM 1 (48.1 KB DOCX)

## Data Availability

The datasets and analysis code generated and/or analyzed during the current study are available from the corresponding author, Sumeet Singh-Tan (ssinght@montefiore.org), on reasonable request and subject.
