# Supplementary material for: Impact of Medication for Opioid Use Disorder on Patient Directed Discharge Among Patients with Opioid Use Disorder
Source: J Gen Intern Med. 2026 Jan 23;41(9):2448–55. doi: 10.1007/s11606-026-10172-5 (PMC13304040; doi:10.1007/s11606-026-10172-5)
Supplement: Supplementary file 1 — (48.1 KB DOCX) [file 11606_2026_10172_MOESM1_ESM.docx]

Supplementary Content

Supplement 1: Diagnosis Codes

- Opioid Use Disorder 2
- Mental Health Diagnoses 3
- Substance Use Disorders 3
- Infections 4
- Liver Disease 9

Supplement 2: Dates of exclusion from study period due to COVID-19 11

Supplement 3: Methadone Dose thresholds for study inclusion 12

**Supplement 1: Diagnosis Codes**

OUD

Poisoning (0)

| \| T40.1X1A \| \| --- \| \| T40.1X1D \| \| T40.1X3A \| \| T40.1X2A \| \| T40.1X4A \| \| T40.3X1A \| \| T40.3X2A \| \| T40.3X3A \| \| T40.3X4A \| \| T40.0X1A \| \| T40.0X2A \| \| T40.2X1A \| \| T40.2X1D \| \| T40.2X2A \| \| T40.2X2D \| \| T40.2X4A \| \| T40.2X4D \| \| T40.4X1A \| \| T40.4X2A \| \| T40.4X4A \| \| T40.601A \| \| T40.602A \| \| T40.603A \| \| T40.604A \| | \| Poisoning by heroin, accidental (unintentional), initial encounter \| \| --- \| \| Poisoning by heroin, accidental (unintentional), subsequent encounter \| \| Poisoning by heroin, assault, initial encounter \| \| Poisoning by heroin, intentional self-harm, initial encounter \| \| Poisoning by heroin, undetermined, initial encounter \| \| Poisoning by methadone, accidental (unintentional), initial encounter \| \| Poisoning by methadone, intentional self-harm, initial encounter \| \| Poisoning by methadone, assault, initial encounter \| \| Poisoning by methadone, undetermined, initial encounter \| \| Poisoning by opium, accidental (unintentional), initial encounter \| \| Poisoning by opium, intentional self-harm, initial encounter \| \| Poisoning by other opioids, accidental (unintentional), initial encounter \| \| Poisoning by other opioids, accidental (unintentional), subsequent encounter \| \| Poisoning by other opioids, intentional self-harm, initial encounter \| \| Poisoning by other opioids, intentional self-harm, subsequent encounter \| \| Poisoning by other opioids, undetermined, initial encounter \| \| Poisoning by other opioids, undetermined, subsequent encounter \| \| Poisoning by other synthetic narcotics, accidental (unintentional), initial encounter \| \| Poisoning by other synthetic narcotics, intentional self-harm, initial encounter \| \| Poisoning by other synthetic narcotics, undetermined, initial encounter \| \| Poisoning by unspecified narcotics, accidental (unintentional), initial encounter \| \| Poisoning by unspecified narcotics, intentional self-harm, initial encounter \| \| Poisoning by unspecified narcotics, assault, initial encounter \| \| Poisoning by unspecified narcotics, undetermined, initial encounter \| |
| --- | --- | --- | --- | --- | --- | --- | --- | --- | --- | --- | --- | --- | --- | --- | --- | --- | --- | --- | --- | --- | --- | --- | --- | --- | --- | --- | --- | --- | --- | --- | --- | --- | --- | --- | --- | --- | --- | --- | --- | --- | --- | --- | --- | --- | --- | --- | --- | --- | --- |

Opioid Abuse & Opioid-Induced Disorder (1)

| F11.10  F11.14  F11.19  F11.120  F11.121  F11.122  F11.129  F11.150  F11.151  F11.159  F11.188  F11.29  F11.288  F11.99  F11.988 | Opioid abuse, uncomplicated  Opioid abuse with opioid induced mood disorder  Opioid abuse with unspecified opioid induced disorder  Opioid abuse with intoxication, uncomplicated  Opioid abuse with intoxication delirium  Opioid abuse with intoxication with perceptual disturbance  Opioid abuse with intoxication, unspecified  Opioid abuse with opioid-induced psychotic disorder with delusions  Opioid abuse with opioid induced psychotic disorder with hallucinations  Opioid abuse with opioid induced psychotic disorder, unspecified  Opioid abuse with other opioid induced disorder  Opioid dependence with unspecified opioid-induced disorder  Opioid dependence with other opioid-induced disorder  Opioid use, unspecified with unspecified opioid-induced disorder  Opioid use, unspecified with other opioid-induced disorder |
| --- | --- |

Opioid Use or Opioid Dependence with Indication of Current Complication (2)

| F11.23  F11.24  F11.220  F11.221  F11.222  F11.229  F11.251  F11.259  F11.282  F11.93  F11.94  F11.920  F11.921  F11.929  F11.951  F11.959  F11.982 | Opioid dependence with withdrawal  Opioid dependence with opioid-induced mood disorder  Opioid dependence with intoxication, uncomplicated  Opioid dependence with intoxication delirium  Opioid dependence with intoxication with perceptual disturbance  Opioid dependence with intoxication, unspecified  Opioid dependence with opioid induced psychotic disorder with hallucinations  Opioid dependence with opioid induced psychotic disorder, unspecified  Opioid dependence with opioid induced sleep disorder  Opioid use, unspecified with withdrawal  Opioid use, unspecified with opioid induced mood disorder  Opioid use, unspecified with intoxication, uncomplicated  Opioid use, unspecified with intoxication delirium  Opioid use, unspecified with intoxication, unspecified  Opioid use, unspecified with opioid induced psychotic disorder with hallucinations  Opioid use, unspecified with opioid induced psychotic disorder, unspecified  Opioid use, unspecified with opioid induced sleep disorder |
| --- | --- |

Adverse Effect (3)

| \| T40.3X5A \| \| --- \| \| T40.0X5A \| \| T40.695A \| \| T40.2X5A \| \| T40.2X5D \| \| T40.4X5A \| \| T40.605A \| | \| Adverse effect of methadone, initial encounter \| \| --- \| \| Adverse effect of opium, initial encounter \| \| Adverse effect of other narcotics, initial encounter \| \| Adverse effect of other opioids, initial encounter \| \| Adverse effect of other opioids, subsequent encounter \| \| Adverse effect of other synthetic narcotics, initial encounter \| \| Adverse effect of unspecified narcotics, initial encounter \| |
| --- | --- | --- | --- | --- | --- | --- | --- | --- | --- | --- | --- | --- | --- | --- | --- |

Opioid Use or Opioid Dependence without Indication of Current Complication (4)

| F11.20  F11.21  F11.90  Z79.891 | Opioid dependence, uncomplicated  Opioid dependence, in remission  Opioid use, unspecified, uncomplicated  Long term (current) use of opiate analgesic |
| --- | --- |

Mental Health Diagnoses

Anxiety Disorder:

F411, F413, F418, F419

Schizophrenia/Schizoaffective Disorder:

F200, F201, F202, F203, F2089, F209, F21, F22, F23, F24, F250, F251, F258, F259

Bipolar Disorder:

F3010, F3011, F3012, F3013, F302, F303, F304, F308, F309, F309, F310, F3110, F3111, F3112, F3113, F312, F3130, F3131, F3132, F314, F315, F3160, F3161, F3162, F3163, F3164, F3170, F3171, F3173, F3175, F3177, F3181, F3189, F319

Depression:

F320, F321, F322, F323, F324, F328, F32A, F330, F331, F332, F333, F3340, F3341, F3342, F338, F339, F348, F3481, F3489, F349, F39

PTSD:

F4310, F4311, F4312, F4320, F4321, F4322, F4323

Personality Disorder:

F600, F601, F602, F603, F604, F605, F606, F607, F6081, F6089, F609

Comorbid Substance Use Disorders

Sedative, Hypnotic, or Anxiolytic Abuse/Dependence/Use:

F1310, F13120, F13129, F1320, F13220, F13229, F13230, F13239, F1390, F13920, F13921, F13929, F13930, F13939

Cocaine Abuse/Dependence/Use:

F1410, F14120, F14129, F1420, F14220, F14229, F1423, F1490, F14920, F14929

Stimulant Abuse/Dependence/Use:

F1510, F15120, F15129, F1520, F15220, F15229, F1523, F1590, F15920, F15929, F1593

Alcohol Abuse/Dependence/Use:

F1014, F1024, F10151, F10250, F10251, F10159, F10259, F10180, F10188, F1019, F1026, F1027, F10280, F10282, F10288, F1029, F10231, F10232, F10230, F10239, F10120, F10129, F10220, F10229, F10121, F10221, F1010, F1020, F1021

Infections

Endocarditis

| I33 | Acute and subacute infective endocarditis |
| --- | --- |
| I38 | Endocarditis, valve unspecified |
| I339 | Acute and subacute infective endocarditis, unspecified |
| T826 | Infection and inflammatory reaction due to cardiac valve prosthesis |
| B376 | Candidal endocarditis |
| I39 (1390) | Endocarditis and heart valve disorders in disease classified elsewhere |
| I398 | Endocarditis, valve unspecified, in disease classified elsewhere |

Bacteremia

| R78.81 | Bacteremia |
| --- | --- |

Cellulitis

| L03.0 | Cellulitis and acute lymphangitis of finder and toe |
| --- | --- |
| L03.01 | Cellulitis of finger |
| L03.011 | Cellulitis of right finger |
| L03.012 | Cellulitis of left finger |
| L03.019 | Cellulitis of unspecified finger |
| L03.03 | Cellulitis of toe |
| L03.031 | Cellulitis of right toe |
| L03.032 | Cellulitis of left toe |
| L03.039 | Cellulitis of unspecified toe |
| L03.1 | Cellulitis and acute lymphangitis of other parts of limb |
| L03.11 | Cellulitis of other parts of limb |
| L03.111 | Cellulitis of right axilla |
| L03.112 | Cellulitis of left axilla |
| L03.113 | Cellulitis of right upper limb |
| L03.114 | Cellulitis of left upper limb |
| L03.115 | Cellulitis of right lower limb |
| L03.116 | Cellulitis of left lower limb |
| L03.119 | Cellulitis of unspecified part of limb |
| L03.2 | Cellulitis and acute lymphangitis of face and neck |
| L03.21 | Cellulitis and acute lymphangitis of face |
| L03.211 | Cellulitis of face |
| L03.213 | Periorbital cellulitis |
| L03.22 | Cellulitis and acute lymphangitis of neck |
| L03.221 | Cellulitis of neck |
| L03.3 | Cellulitis and acute lymphangitis of trunk |
| L03.31 | Cellulitis of trunk |
| L03.311 | Cellulitis of abdominal wall |
| L03.312 | Cellulitis of back [any part except buttock] |
| L03.313 | Cellulitis of chest wall |
| L03.314 | Cellulitis of groin |
| L03.315 | Cellulitis of perineum |
| L03.316 | Cellulitis of umbilicus |
| L03.317 | Cellulitis of buttock |
| L03.319 | Cellulitis of trunk, unspecified |
| L03.8 | Cellulitis and acute lymphangitis of other sites |
| L03.81 | Cellulitis of other sites |
| L03.811 | Cellulitis of head [any part, except face] |
| L03.818 | Cellulitis of other sites |
| L03.9 | Cellulitis and acute lymphangitis, unspecified |
| L03.90 | Cellulitis, unspecified |

Osteomyelitis

| M86 | Osteomyelitis |
| --- | --- |
| M86.0 | Acute hematogenous osteomyelitis |
| M86.00 | Acute hematogenous osteomyelitis, unspecified site |
| M86.01 | Acute hematogenous osteomyelitis, shoulder |
| M86.011 | Acute hematogenous osteomyelitis, right shoulder |
| M86.012 | Acute hematogenous osteomyelitis, left shoulder |
| M86.019 | Acute hematogenous osteomyelitis, unspecified shoulder |
| M86.02 | Acute hematogenous osteomyelitis, humerus |
| M86.021 | Acute hematogenous osteomyelitis, right humerus |
| M86.022 | Acute hematogenous osteomyelitis, left humerus |
| M86.029 | Acute hematogenous osteomyelitis, unspecified humerus |
| M86.03 | Acute hematogenous osteomyelitis, radius and ulna |
| M86.031 | Acute hematogenous osteomyelitis, right radius and ulna |
| M86.032 | Acute hematogenous osteomyelitis, left radius and ulna |
| M86.039 | Acute hematogenous osteomyelitis, unspecified radius and ulna |
| M86.04 | Acute hematogenous osteomyelitis, hand |
| M86.041 | Acute hematogenous osteomyelitis, right hand |
| M86.042 | Acute hematogenous osteomyelitis, left hand |
| M86.049 | Acute hematogenous osteomyelitis, unspecified hand |
| M86.05 | Acute hematogenous osteomyelitis, femur |
| M86.051 |  |
| M86.052 |  |
| M86.059 |  |
| M86.06 | Acute hematogenous osteomyelitis, tibia and fibula |
| M86.061 |  |
| M86.062 |  |
| M86.069 |  |
| M86.07 | Acute hematogenous osteomyelitis, ankle and foot |
| M86.071 |  |
| M86.072 |  |
| M86.079 |  |
| M86.08 | Acute hematogenous osteomyelitis, other sites |
| M86.09 | Acute hematogenous osteomyelitis, multiple site |
| M86.1 | Other acute osteomyelitis |
| M86.10 | Other acute osteomyelitis, unspecified site |
| M86.11 | Other acute osteomyelitis, shoulder |
| M86.111 | Other acute osteomyelitis, right shoulder |
| M86.112 | Other acute osteomyelitis, left shoulder |
| M86.119 | Other acute osteomyelitis, unspecified shoulder |
| M86.12 | Other acute osteomyelitis, humerus |
| M86.121 |  |
| M86.122 |  |
| M86.129 |  |
| M86.13 | Other acute osteomyelitis, radius and ulna |
| M86.131 |  |
| M86.132 |  |
| M86.139 |  |
| M86.14 | Other acute osteomyelitis, hand |
| M86.141 |  |
| M86.142 |  |
| M86.149 |  |
| M86.15 | Other acute osteomyelitis, femur |
| M86.151 |  |
| M86.152 |  |
| M86.159 |  |
| M86.16 | Other acute osteomyelitis, tibia and fibula |
| M86.161 |  |
| M86.162 |  |
| M86.169 |  |
| M86.17 | Other acute osteomyelitis, ankle and foot |
| M86.171 |  |
| M86.172 |  |
| M86.179 |  |
| M86.18 | Other acute osteomyelitis, other site |
| M86.19 | Other acute osteomyelitis, multiple sites |
| M86.2 | Subacute osteomyelitis |
| M86.20 | Subacute osteomyelitis, unspecified site |
| M86.21 | Subacute osteomyelitis, shoulder |
| M86.211 |  |
| M86.212 |  |
| M86.219 |  |
| M86.22 | Subacute osteomyelitis, humerus |
| M86.221 |  |
| M86.222 |  |
| M86.229 |  |
| M86.23 | Subacute osteomyelitis, radius and ulna |
| M86.231 |  |
| M86.232 |  |
| M86.239 |  |
| M86.24 | Subacute osteomyelitis, hand |
| M86.241 |  |
| M86.242 |  |
| M86.249 |  |
| M86.25 | Subacute osteomyelitis, femur |
| M86.251 |  |
| M86.252 |  |
| M86.259 |  |
| M86.26 | Subacute osteomyelitis, tibia and fibula |
| M86.261 |  |
| M86.262 |  |
| M86.269 |  |
| M86.27 | Subacute osteomyelitis, ankle and foot |
| M86.271 |  |
| M86.272 |  |
| M86.279 |  |
| M86.28 | Subacute osteomyelitis, other site |
| M86.29 | Subacute osteomyelitis, multiple sites |
| M86.3 | Chronic multifocal osteomyelitis |
| M86.30 | Chronic multifocal osteomyelitis, unspecified site |
| M86.31 | Chronic multifocal osteomyelitis, shoulder |
| M86.311 |  |
| M86.312 |  |
| M86.319 |  |
| M86.32 | Chronic multifocal osteomyelitis, humerus |
| M86.321 |  |
| M86.322 |  |
| M86.329 |  |
| M86.33 | Chronic multifocal osteomyelitis, radius and ulna |
| M86.331 |  |
| M86.332 |  |
| M86.339 |  |
| M86.34 | Chronic multifocal osteomyelitis, hand |
| M86.341 |  |
| M86.342 |  |
| M86.349 |  |
| M86.35 | Chronic multifocal osteomyelitis, femur |
| M86.351 |  |
| M86.352 |  |
| M86.359 |  |
| M86.36 | Chronic multifocal osteomyelitis, tibia and fibula |
| M86.361 |  |
| M86.362 |  |
| M86.369 |  |
| M86.37 | Chronic multifocal osteomyelitis, ankle and foot |
| M86.371 |  |
| M86.372 |  |
| M86.379 |  |
| M86.38 | Chronic multifocal osteomyelitis, other site |
| M86.389 | Chronic multifocal osteomyelitis, multiple site |
| M86.4 | Chronic osteomyelitis with draining sinus |
| M86.40 | Chronic osteomyelitis with draining sinus, unspecified site |
| M86.41 | Chronic osteomyelitis with draining sinus, shoulder |
| M86.411 |  |
| M86.412 |  |
| M86.419 |  |
| M86.42 | Chronic osteomyelitis with draining sinus, humerus |
| M86.421 |  |
| M86.422 |  |
| M86.429 |  |
| M86.43 | Chronic osteomyelitis with draining sinus, radius and ulna |
| M86.431 |  |
| M86.432 |  |
| M86.439 |  |
| M86.44 | Chronic osteomyelitis with draining sinus, hand |
| M86.441 |  |
| M86.442 |  |
| M86.449 |  |
| M86.45 | Chronic osteomyelitis with draining sinus, femur |
| M86.451 |  |
| M86.452 |  |
| M86.459 |  |
| M86.46 | Chronic osteomyelitis with draining sinus, tibia and fibula |
| M86.461 |  |
| M86.462 |  |
| M86.469 |  |
| M86.47 | Chronic osteomyelitis with draining sinus, ankle and foot |
| M86.471 |  |
| M86.472 |  |
| M86.479 |  |
| M86.48 | Chronic osteomyelitis with draining sinus, other site |
| M86.49 | Chronic osteomyelitis with draining sinus, multiple site |
| M86.5 | Other chronic hematogenous osteomyelitis |
| M86.50 | Other chronic hematogenous osteomyelitis, unspecified site |
| M86.51 | Other chronic hematogenous osteomyelitis, shoulder |
| M86.511 |  |
| M86.512 |  |
| M86.519 |  |
| M86.52 | Other chronic hematogenous osteomyelitis, humerus |
| M86.521 |  |
| M86.522 |  |
| M86.529 |  |
| M86.53 | Other chronic hematogenous oseomyelitis, radius and ulna |
| M86.531 |  |
| M86.532 |  |
| M86.539 |  |
| M86.54 | Other chronic hematogenous osteomyelitis, hand |
| M86.541 |  |
| M86.542 |  |
| M86.549 |  |
| M86.55 | Other chronic hematogenous osteomyelitis, femur |
| M86.551 |  |
| M86.552 |  |
| M86.559 |  |
| M86.56 | Other chronic hematogenous osteomyelitis, tibia and fibula |
| M86.561 |  |
| M86.562 |  |
| M86.569 |  |
| M86.57 | Other chronic hematogenous osteomyelitis, ankle and foot |
| M86.571 |  |
| M86.572 |  |
| M86.579 |  |
| M86.58 | Other chronic hematogenous osteomyelitis, other site |
| M86.59 | Other chronic hematogenous osteomyelitis, multiple sites |
| M86.6 | Other chronic osteomyelitis |
| M86.60 | Other chronic osteomyelitis, unspecified site |
| M86.61 | Other chronic osteomyelitis, shoulder |
| M86.611 |  |
| M86.612 |  |
| M86.619 |  |
| M86.62 | Other chronic osteomyelitis, humerus |
| M86.621 |  |
| M86.622 |  |
| M86.629 |  |
| M86.63 | Other chronic osteomyelitis, radius and ulna |
| M86.631 |  |
| M86.632 |  |
| M86.639 |  |
| M86.64 | Other chronic osteomyelitis, hand |
| M86.641 |  |
| M86.642 |  |
| M86.649 |  |
| M86.65 | Other chronic osteomyelitis, thigh |
| M86.651 |  |
| M86.652 |  |
| M86.659 |  |
| M86.66 | Other chronic osteomyelitis, tibia and fibua |
| M86.661 |  |
| M86.662 |  |
| M86.669 |  |
| M86.67 | Other chronic osteomyelitis, ankle and foot |
| M86.671 |  |
| M86.672 |  |
| M86.679 |  |
| M86.68 | Other chronic osteomyelitis, other site |
| M86.69 | Other chronic osteomyelitis, multiple sites |
| M86.8X | Other osteomyelitis |
| M86.8X0 | Other osteomyelitis, multiple sites |
| M86.8X1 | Other osteomyelitis, shoulder |
| M86.8X2 | Other osteomyelitis, upper arm |
| M86.8X3 | Other osteomyelitis, forearm |
| M86.8X4 | Other osteomyelitis, hand |
| M86.8X5 | Other osteomyelitis, thigh |
| M86.8X6 | Other osteomyelitis, lower leg |
| M86.8X7 | Other osteomyelitis, ankle and foot |
| M86.8X8 | Other osteomyelitis, other site |
| M86.8X9 | Other osteomyelitis, unspecified sites |
| M89.9 | Osteomyelitis, unspecified |
| M46.40 | Discitis |
| M46.41 | Occipito-atlanto-axial discitis |
| M46.42 | Cervical discitis |
| M46.43 | Cervicothoracic discitis |
| M46.44 | Thoracic discitis |
| M46.45 | Thoracolumbar discitis |
| M46.46 | Lumbar discitis |
| M46.47 | Lumbosacral discitis |
| M46.48 | Sacrococcygeal discitis |
| M46.49 | Discitis of multiple sites |

Liver Disease

| K70.00 | Alcoholic fatty liver |
| --- | --- |
| K70.10 | Alcoholic hepatitis, without ascites |
| K70.11 | Alcoholic hepatitis, with ascites |
| K70.20 | Alcoholic fibrosis and sclerosis of liver |
| K70.30 | Alcoholic cirrhosis of the liver without ascites |
| K70.31 | Alcohol cirrhosis of the liver with ascites |
| K70.40 | Alcoholic hepatic failure without coma |
| K70.41 | Alcoholic hepatic failure with coma |
| K70.90 | Alcoholic liver disease, unspecified |
| B15.00 | Acute hepatitis A with hepatic coma |
| B15.90 | Acute hepatitis A without hepatic coma |
| B16.00 | Acute hepatitis B with delta agent with hepatic coma |
| B16.10 | Acute hepatitis B with delta agent without hepatic coma |
| B16.20 | Acute hepatitis B without delta agent with hepatic coma |
| B16.90 | Acute hepatitis B without delta agent without hepatic coma |
| B17.00 | Acute delta-super infection with hepatitis B carrier |
| B17.10 | Acute hepatitis C without hepatic coma |
| B17.11 | Acute hepatitis S with hepatic coma |
| B17.20 | Acute hepatitis E |
| B17.80 | Other specified acute viral hepatitis |
| B17.90 | Acute viral hepatitis, unspecified |
| B18.00 | Chronic viral hepatitis B with delta-agent |
| B18.10 | Chronic viral hepatitis B with delta agent |
| B18.10 | Chronic viral hepatitis B without delta agent |
| B18.20 | Chronic viral hepatitis C |
| B18.80 | Other chronic viral hepatitis |
| B18.90 | Chronic viral hepatitis, unspecified |
| B19.00 | Unspecified viral hepatitis with hepatic coma |
| B19.10 | Unspecified vital hepatitis B without hepatic coma |
| B19.11 | Unspecified vital hepatitis B with hepatic coma |
| B19.20 | Unspecified viral hepatitis C without hepatic coma |
| B19.21 | Unspecified viral hepatitis C with hepatic coma |
| B19.90 | Unspecified viral hepatitis without hepatic coma |
| R74.01 | Elevation of levels of liver transaminase levels (ALT, AST) |
| R94.50 | Abnormal results of liver function studies |

**Supplement 2: COVID-19 Exclusion Dates**

March 1, 2020 – May 31, 2020

December 1, 2020 – March 31, 2021

December 1, 2021 – January 31, 2022

**Supplement 3: Methadone dosing cutoffs**

A post hoc sensitivity analysis was completed to evaluate the dose thresholds of methadone for inclusion:

| **60mg Methadone*** | | | | |
| --- | --- | --- | --- | --- |
|  | Inpatient MOUD  (n=2796) | Matched Control (n=2796) | p-value | Odds Ratio (CI) |
| Patient Directed Discharge | 324 (11.6%) | 377 (13.5%) | 0.03 | 0.84 (0.70-1.00) |
| **40mg Methadone (current definition)^ß^** | | | | |
|  | Inpatient MOUD  (n=2771) | Matched Control  (n=2771) | p-value | Odds Ratio (CI) |
| Patient Directed Discharge | 329 (11.9%) | 399 (14.4%) | 0.005 | 0.80 (0.67-0.96) |
| **20mg Methadone^ƒ^** | | | | |
|  | Inpatient MOUD  (n=1437) | Matched Control (n=1437) | p-value | Odds Ratio (CI) |
| Patient Directed Discharge | 170 (11.8%) | 189 (13.2%) | 0.28 | 0.89 (0.70-1.12) |
| *Inclusion criteria: Day 2 methadone dose ≤60mg OR no evidence of enrollment at a Montefiore affiliated treatment site within the prior 60days OR day 1 buprenorphine dose >2mg OR no buprenorphine prescription within the prior 60 days  ß Inclusion criteria: Day 2 methadone dose ≤40mg OR no evidence of enrollment at a Montefiore affiliated treatment site within the prior 60days OR day 1 buprenorphine dose ≤2mg OR no buprenorphine prescription within the prior 60 days  ƒ Inclusion criteria: Day 2 methadone dose ≤20mg OR no evidence of dispensation appointment at a Montefiore affiliated treatment site within the prior 60days OR day 1 buprenorphine dose ≤2mg OR no buprenorphine prescription within the prior 60 days | | | | |
